# Titanium Dioxide Nanoparticles Induce Endoplasmic Reticulum Stress-Mediated Autophagic Cell Death via Mitochondria-Associated Endoplasmic Reticulum Membrane Disruption in Normal Lung Cells

**DOI:** 10.1371/journal.pone.0131208

**Published:** 2015-06-29

**Authors:** Kyeong-Nam Yu, Seung-Hee Chang, Soo Jin Park, Joohyun Lim, Jinkyu Lee, Tae-Jong Yoon, Jun-Sung Kim, Myung-Haing Cho

**Affiliations:** 1 Laboratory of Toxicology, BK21 PLUS Program for Creative Veterinary Science Research, Research Institute for Veterinary Science and College of Veterinary Medicine, Seoul National University, Gwanak-gu, Seoul, Korea; 2 R&D Center, Biterials Co., Siksa-dong, Ilsandong-gu, Goyang-si, Gyeonggi-do, Korea, Korea; 3 Department of Chemistry, College of Natural Sciences, Gwanak-gu, Seoul National University, Seoul, Korea; 4 Department of Applied Bioscience, College of Life Science, CHA University, Pocheon-shi, Gyeonggi-do, Korea; 5 Graduate Group of Tumor Biology, Seoul National University, Gwanak-gu, Seoul, Korea; 6 Graduate School of Convergence Science and Technology, Seoul National University, Yeongtong-Gu, Suwon, Gyeonggi-Do, Korea; 7 Advanced Institute of Convergence Technology, Seoul National University, Suwon, Gyeonggi-Do, Korea; Hokkaido University, JAPAN

## Abstract

Nanomaterials are used in diverse fields including food, cosmetic, and medical industries. Titanium dioxide nanoparticles (TiO_2_-NP) are widely used, but their effects on biological systems and mechanism of toxicity have not been elucidated fully. Here, we report the toxicological mechanism of TiO_2_-NP in cell organelles. Human bronchial epithelial cells (16HBE14o-) were exposed to 50 and 100 μg/mL TiO_2_-NP for 24 and 48 h. Our results showed that TiO_2_-NP induced endoplasmic reticulum (ER) stress in the cells and disrupted the mitochondria-associated endoplasmic reticulum membranes (MAMs) and calcium ion balance, thereby increasing autophagy. In contrast, an inhibitor of ER stress, tauroursodeoxycholic acid (TUDCA), mitigated the cellular toxic response, suggesting that TiO_2_-NP promoted toxicity via ER stress. This novel mechanism of TiO_2_-NP toxicity in human bronchial epithelial cells suggests that further exhaustive research on the harmful effects of these nanoparticles in relevant organisms is needed for their safe application.

## Introduction

Titanium dioxide nanoparticles (TiO_2_-NP) have widespread applications in various fields and are used in sunscreens, cosmetics, food products, toothpastes, and medical reagents [1–4]. In recent years, many studies have focused on the biomedical application of TiO_2_-NP in areas such as cancer therapy, drug delivery systems, cell imaging, genetic engineering, biosensors, and biological experiments [5–7].

However, with the increasing developments in the application of TiO_2_-NP, concerns regarding their toxicity to humans also increase. Many studies have reported that TiO_2_-NP elicit a toxic response in *in vitro* and *in vivo* systems. Bhattacharya et al. reported that TiO_2_-NP of <100 nm in diameter were able to generate free radicals and elevate DNA adduct formation (8-OHdG) in human lung fibroblasts [8]. In addition, in A549 cells, the anatase TiO_2_-NP induced mitochondrial injury in a dose-dependent manner owing to reactive oxygen species (ROS) generation [9]. Oesch and Landsiedel reviewed the genotoxicity of TiO_2_-NP using various test results [10]. Moreover, Sager et al. reported that P-25 TiO_2_-NP suspension (anatase: rutile = 80:20, 21 nm) induces an inflammation response in rats [11]. Oberdorster et al. [12] reported a similar result that 21-nm TiO_2_-NP had inflammatory effects on the alveolar interstitium in the lungs. Ferin et al. detected polymorphonuclear (PMN) leukocytes in lavage cells in rat lung after inhalation of ~20-nm TiO_2_-NP [13]. Although there are many toxicity results, the detailed molecular mechanism of TiO_2_-NP toxicity is not clear.

The endoplasmic reticulum (ER) is an organelle that regulates protein secretion, cell surface development, and maintenance of the calcium ion (Ca^2+^) concentration of cells [4]. Thus, disruption of ER homeostasis leads to protein misfolding and ER stress, which affect both the quality control and translation of protein. The membranes of the ER and mitochondria are enriched with Ca^2+^-binding chaperones called mitochondria-associated ER membranes (MAMs), which preserve and regulate cellular homeostasis in different environments [14]. Studies have shown that ER stress is linked closely to changes in the composition of MAMs, deregulated Ca^2+^ transport, and cell death [15]. Furthermore, ER stress is associated with protein degradation *via* autophagy, which at abnormal levels, leads to cytotoxic processing or mechanisms such as apoptosis [16].

In this study, we demonstrated that ER stress-mediated MAM disruption, autophagy, and mitochondrial dysfunction might play a key role in the TiO_2_-NP-induced toxic responses in human bronchial epithelial cells.

## Materials and Methods

### Characterization of TiO_2_ nanoparticles

The TiO_2_ nanoparticles (TiO_2_-NP, P-25; anatase:rutile, 8:2) were purchased from Degussa Korea (Inchon, Korea). The structure and morphology of the TiO_2_-NP were characterized by transmission electron microscopy (TEM) with an accelerating voltage of 100 kV. The TEM samples were dispersed in methanol, and a drop of the suspension was placed on formvar-carbon film on a square 300-mesh copper grid, followed by drying the grid at room temperature for 1 h. We conducted X-ray diffraction (XRD) using the X’pert PW1827 diffractometer (Philips, Netherlands) to confirm the crystal structure of the TiO_2_-NP [17]. The goniometer was motorized and moved through a scanning range of θ–2θ. The diffractometer was operated at 40 kV and 40 mA in the range of 20–80°. The steps were performed in increments of 0.05°, and counts were collected for 5 s at each step [18]. For dynamic light scattering (DLS) measurements, 4 mL of a 0.2 mg/mL suspension of TiO_2_-NPs in distilled water was sonicated for 30 s. The hydrodynamic sizes and zeta potentials of the particle suspension were measured at room temperature using an Electrophoretic Light Scattering Spectrophotometer (ELS-8000, Photal, Osaka, Japan), with an accumulation time of 70 times and an equilibration time of 60 s.

### Suspension of TiO_2_ nanoparticles

We chose a suspension protocol that has been proven to yield the best dispersion of the nanomaterials in previous research [19]. For suspension in culture medium, TiO_2_-NP powder was dispersed in phosphate-buffered saline (PBS) at 10 mg/mL and sonicated for 10 min using an Ultrasonic cleaner (5510-DTH, Branson, MI, USA). After sonication, to prepare the end-point concentrations, Dulbecco’s modified Eagle's medium (DMEM)-F12 medium (Gibco, NY, USA) was transferred to test tubes and diluted with the TiO_2_-NP stock solution.

### Cell culture and viability assay

The human bronchial epithelial cells (16HBE14o-) were a gift from Dr. Dieter Gruenert (University of California, San Francisco, CA, USA). The cells were incubated in DMEM-F12 medium (Gibco) supplemented with 5% fetal bovine serum (FBS) and 1% penicillin-streptomycin. Cell viability and proliferation were determined following treatment with TiO_2_-NP using the xCELLigence RTCA DP system (Roche, Basel, Switzerland), which monitors cellular events in real-time without incorporated labels [17]. Briefly, cells (0.5 × 10^3^) were seeded in each chamber of an E-plate. After an overnight incubation, cells were treated with 25, 50, and 100 μg/mL TiO_2_-NPs and monitored for 72 h. This system observes electrical impedance across interdigitated microelectrodes that are integrated on the bottom of the E-plate. The detection of impedance provides quantitative results about the cell number, proliferation, and viability. To measure the potential effects of TiO_2_-NP on cells, xCELLigence RTCA DP system-based cell viability assay was performed. Briefly, after seeding the cells (0.5x10^4^) in 96-well plates, MTT assay using the reagent MTT [3-(4,5-dimethylthiazol-2-yl)-2,5-diphenyltetrazolium bromide] (Sigma, MA, USA) and WST-1 assay using reagent WST-1 [2-(4-iodophenyl)-3-(4-nitrophenyl)-5-(2,4-disulfophenyl)-2H-tetrazolium] (Roche, Basel, Switzerland) were performed after 24h and 48h of TiO_2_-NP treatment. All experiments were repeated three times.

### Measurement of reactive oxygen species (ROS)

The generation of ROS in the cells following TiO_2_-NP treatment was determined using 2,7-dichlorodihydrofluorescein diacetate (H_2_-DCFDA), an indicator of intracellular ROS [20]. The process involves elimination of the acetate groups (non-fluorescent) by intracellular esterases with the generation of oxidation in the cells [21]. H_2_-DCFDA stock solution (10 mM) in DMSO was diluted in medium to attain a 5 μM working concentration. After incubation for 1 h, the cells were rinsed with PBS and fixed with 4% paraformaldehyde for 10 min at room temperature. The fluorescence was detected using a confocal laser scanning microscope (LSM710, Carl Zeiss, Germany) and fluorescence-activated cells sorting (FACS, BD Bioscience, New Jersey, USA) [17].

### Western blot assay

After homogenization of the cells, the protein concentration was measured using the Bradford protein assay (Bio-Rad, CA, USA). Protein (25 μg) was loaded into the wells, separated on sodium dodecyl sulfate (SDS)-polyacrylamide gel electrophoresis (PAGE) gels (10–15%) (80 V, 3 h), and transferred to a nitrocellulose membrane (50 V, 2 h). After membranes were blocked in 5% skim milk for 1 h, immunoblotting was conducted by incubating with the primary antibodies overnight at 4°C. In this study, 78-kDa glucose-regulated protein (Grp78), binding immunoglobulin protein (Bip), inositol-requiring protein 1 (IRE-1), phospho-IRE-1α, C/EBP homologous protein (CHOP), and microtubule-associated protein-1 light-chain 3 (LC3) antibodies were purchased from Cell Signaling (MA, USA). Sequestosome 1 (SQSTM1, p62), voltage-dependent anion-selective channel protein 1 (VDAC1), and beclin-1 (BECN1) antibodies were purchased from Abcam (MA, USA). Actin and 75 kDa glucose regulated protein (Grp75) antibodies were purchased from Santa Cruz (CA, USA). After incubation with HRP-conjugated secondary antibodies for 2 h at room temperature and washing, the bands of interest were measured using an ATTO CS image analyzer 3.0 (ATTO Corp., Tokyo, Japan) [17].

### Immunofluorescence staining

The TiO_2_-NP-treated cells were fixed with 4% paraformaldehyde (PFA) for 10 min and then treated with 1% Triton X-100 for 15 min. After fixation, the samples were washed and blocked with 3% bovine serum albumin in 1× tris-buffered saline (TBS) for 1 h at 37°C. After washing, samples were incubated overnight with the primary antibodies (IP3R, VDAC, and LC3) diluted 1:200 in blocking solution. After washing with 1× Tween 20-TBS, the fluorescence of the HRP-conjugated secondary antibodies was measured (Invitrogen, CA, USA). After rinsing, samples were mounted with DAKO Cytomation Faramount Aqueous Mounting solution (DAKO, CA, USA) and imaged using confocal laser scanning microscopy (CLSM, LSM710, Carl Zeiss, Germany) [17]. The stained LC3-fluorescent puncta were counted using the Image J program supported by National Institutes of Health (MD, USA).

### Transmission Electron Microscope (TEM) analysis of cellular organelles

The TiO_2_-NP-treated cells were fixed in 2.5% glutaraldehyde with 1% osmium tetroxide (OsO_4_) buffer at 4°C for 6 h. The fixed cells were dehydrated in serially diluted ethanol (30, 50, 70, 80, 90, and 100%). After dehydration, the samples were infiltrated with a 1:1 mixture of propylene oxide and epon at 70°C overnight. Ultrathin sections (about 50 mm) were sliced, mounted on a copper grid, stained with uranyl acetate and lead citrate, and monitored using a JEM 1010 transmission electron microscope (JEOL, Tokyo, Japan) [17].

### Rhod-2 AM staining

Cells (1.0 × 10^4^ cells/well) were seeded on 8-well chamber slides (Nalge Nunc, NY, USA) and incubated for 24 or 48 h with 50 or 100 μg/mL TiO_2_-NP. After incubation, the cells were rinsed with 1 × PBS and incubated with 10 μM Rhod-2 AM ester (Biotium, CA, USA) in PBS for 30 min at 37°C. After 30 min, the cells were washed and fixed with 4% PFA for 15 min at room temperature. The slides were monitored using CLSM (Carl Zeiss, Germany) [21].

### Measurement of mitochondrial intensity and adenosine triphosphate (ATP) levels

After incubation of the cultures with TiO_2_-NP for 24 and 48 h, the mitochondrial intensity was detected by staining with 100 nM Mito-tracker (Invitrogen, CA, USA). The Mito tracker images were analyzed using the Mytoe program, which is software for analyzing mitochondrial dynamics from fluorescence microscope images [22]. To monitor the levels of ATP, normal lung cells were seeded on a 96-well plate and treated with TiO_2_-NP for 24 and 48 h, followed by incubation with 100 μL of CellTiter-Glo reagent (Promega, TN, USA) for 10 min. The luminescent signal was detected using a luminometer (Berthold, Germany).

### Statistical analysis

The results are presented as the mean ± standard error of the mean (SEM) with n = 3. The statistical analyses were carried out using one-way and two-way ANOVA. The differences were considered statically significant with *p* values of *p* < 0.05, *p* < 0.01, and *p* < 0.001.

## Results

### Characterization of TiO_2_ nanoparticles

**Fig 1:**
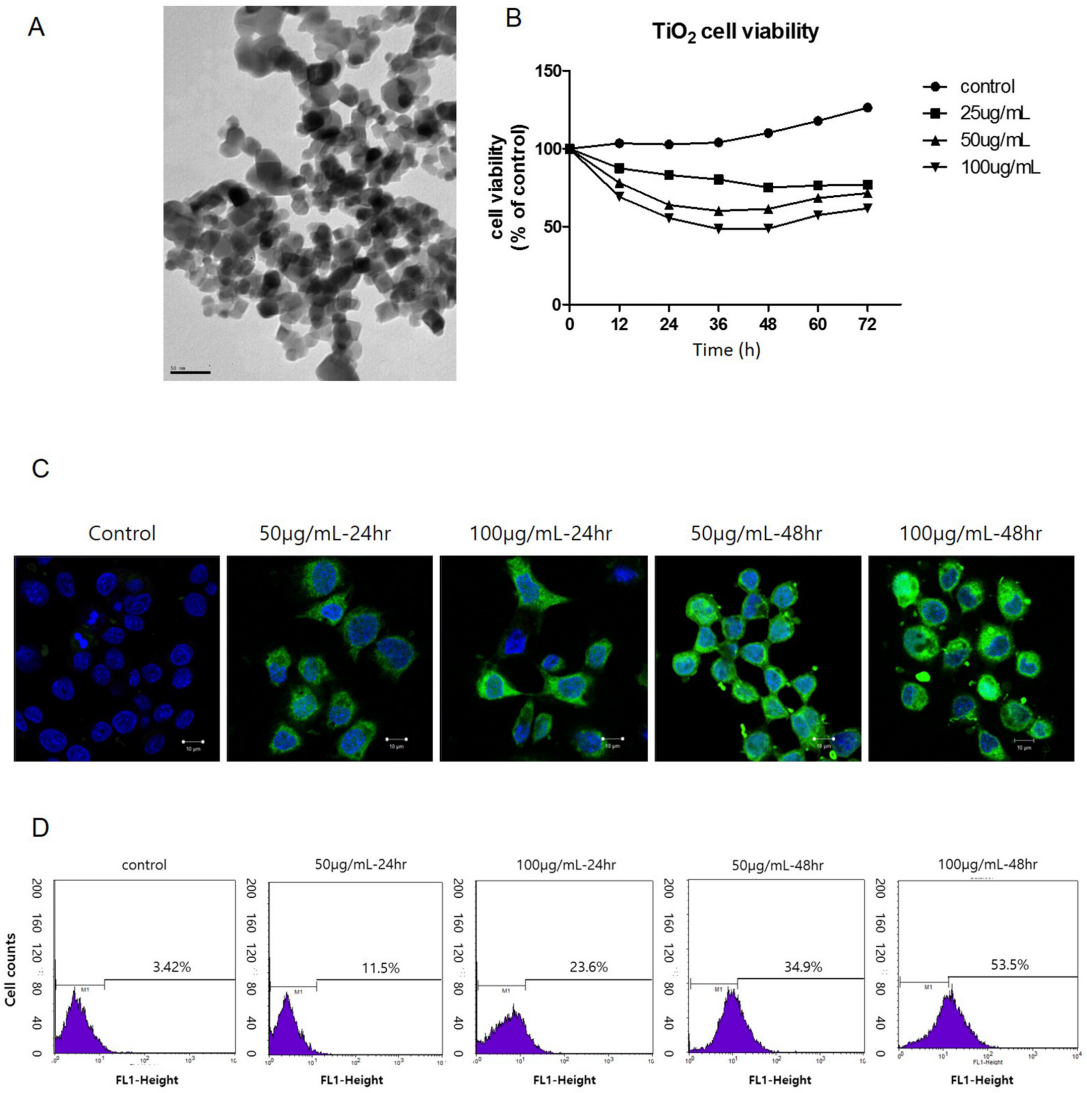
Characterization of titanium dioxide nanoparticles (TiO_2_-NP) and detection of reactive oxygen species (ROS) generation in human bronchial epithelial cells. (A) TEM image of TiO_**2**_-NP shape and size. (B) During exposure of 16HBE14o- cells to TiO_**2**_-NP, viability and proliferation of cells were monitored in real-time using the xCELLigence system for 72 h. (C) To confirm the generation of ROS after TiO_**2**_-NP treatment, H_**2**_-DCFDA staining was performed in cells. The green fluorescence signal was induced gradually (scale bar, 10 μm; magnification, 400×). (D) To quantify the intensity of the green fluorescence, FACS analysis was performed. TEM, transmission electron microscopy; H_**2**_-DCFDA, 2,7-dichlorodihydrofluorescein diacetate; FACS, fluorescence-activated cell sorting.

The particle shape and size of the TiO_2_-NP were analyzed using TEM (Fig 1A). In addition, the XRD pattern showed that the TiO_2_-NP contained a mixture of the anatase and rutile forms (S1 Fig). The hydrodynamic diameter and zeta potential of the TiO_2_-NP were determined using DLS. The TiO_2_-NP had an induced hydrodynamic size of approximately 250 nm in water or in medium, confirming that they are able to aggregate in suspension. The zeta potential results showed that the TiO_2_-NP had negatively charged surfaces in the medium (Table 1). Zeta potential values that are greater than +25 or less than −25 mV typically indicate good stability [23]. Therefore, the zeta potential of TiO_2_-NP in water or medium indicated that the TiO_2_-NP were aggregated in suspension. To investigate the cellular effects of the TiO_2_-NP, cell proliferation and viability were determined during 72 h in real time using the xCELLigence system. The real-time viability of TiO_2_-NP-treated cells was decreased as a function of TiO_2_-NP concentration and time (Fig 1B). We also confirmed this result using the MTT and WST-1 assays. As shown Fig B in S2 Fig, the almost identical cell viability results were obtained from both assays. To evaluate the intracellular ROS generation and elevation induced by TiO_2_-NP, we performed H_2_-DCFDA staining and FACS detection of cells treated with TiO_2_-NP for 24 and 48 h. CLSM images revealed that the fluorescence intensity was proportional to the increased treatment time and concentration of TiO_2_-NP (Fig 1C and Fig A in S2 Fig). According to the FACS data, the H_2_-DCFDA fluorescence increased in TiO_2_-NP treated cells (Fig 1D). Further, the number of stained cells increased in the TiO_2_-NP-treated cells. Our results show that TiO_2_-NP is able to generate intracellular ROS in human bronchial epithelial cells.

**Table 1:** Physical characterization of titanium dioxide nanoparticles (TiO_2_-NP).

	Hydrodynamic diameter (nm)		Zeta potential (mV)	
	Distilled water	Medium	Distilled water	Medium
TiO_2_-NP	220.4	295.9	19.29	-26.01

### TiO_2_ nanoparticles induce ER stress in normal human lung cells

**Fig 2:**
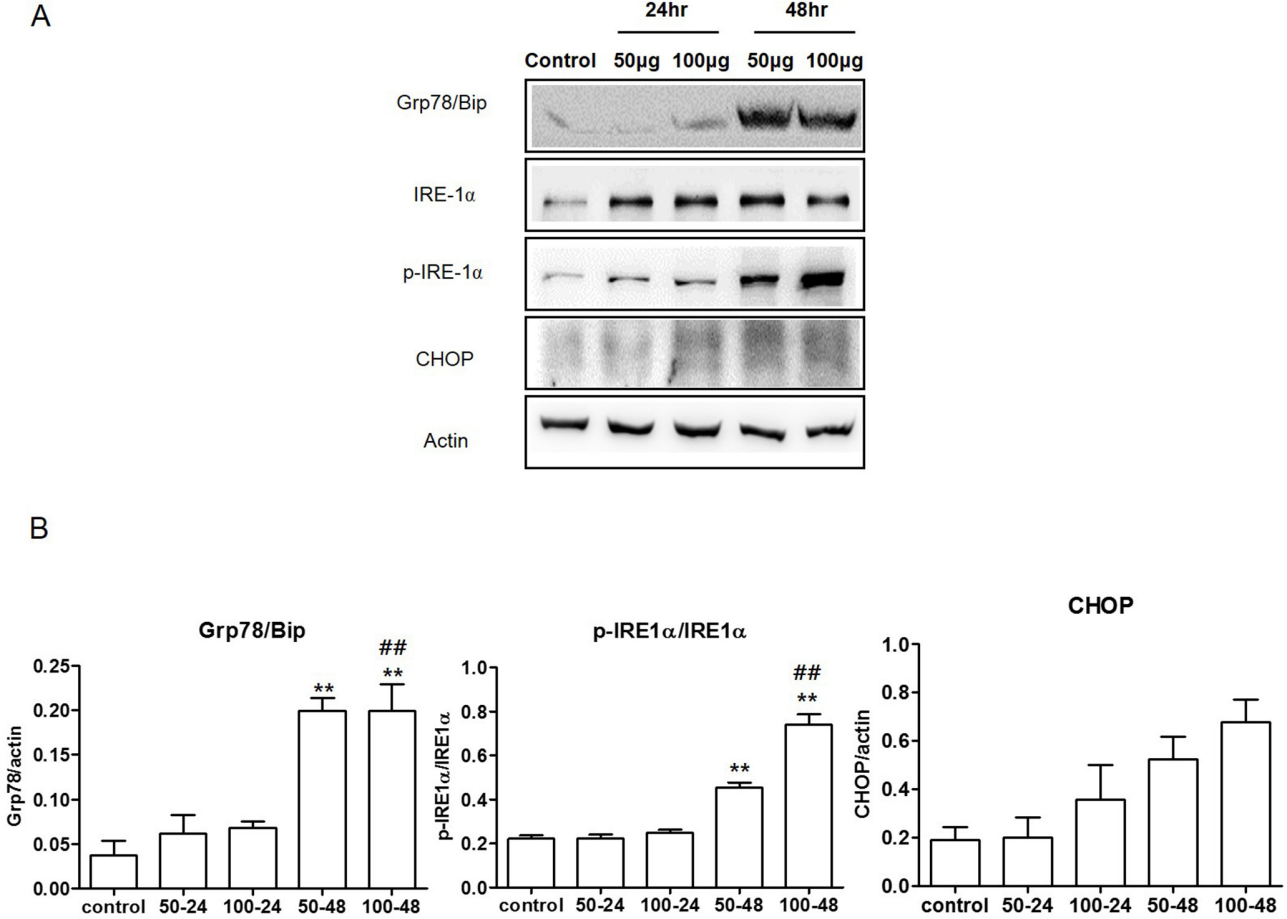
Titanium dioxide nanoparticles (TiO_2_-NP) treatment elevates endoplasmic reticulum (ER) stress in human bronchial epithelial cells. (A) To detect the induction of ER stress after TiO_**2**_-NP treatment, expression levels of Grp78/Bip, IRE-1α, phospho-IRE-1α, and CHOP were detected using western blot analysis. (B) Each bar represents the mean ± SEM, n = 3, **p < 0.01 (comparison between TiO_**2**_-NP concentrations, 50 and 100 μg), ^##^ *p* < 0.01 (comparison between TiO_**2**_-NP treatment times, 24 and 48 h). Grp78, 78-kDa glucose-regulated protein; Bip, binding immunoglobulin protein; phosphorylated inositol-requiring protein 1 (phospho-IRE-1 α); CHOP, C/EBP homologous protein.

To determine the ER stress response in cells following TiO_2_-NP treatment, we incubated human bronchial epithelial cells with various doses of TiO_2_-NP at different time points. The expression of the ER stress-related proteins, including Grp78/Bip, IRE-1α, phospho-IRE-1α, and CHOP, were analyzed by western blot. The results indicated that the expression levels of these proteins increased in TiO_2_-NP-treated cells. The results indicated that the expression levels of these proteins were increased in TiO_2_-NP-treated cells as determined by densitometric analysis (Fig 2A). These results showed that TiO_2_-NP exposure increases ER stress in cells. Phosphorylation of the IRE-1α protein is reported to be a marker of its activation [24]. Therefore, we calculated the ratio of phosphorylated IRE-1α to total IRE-1α. In cells treated with 100 μg/mL TiO_2_-NP for 48 h, the IRE-1α phosphorylation significantly increased. Calnexin is an integral protein of the ER, which is known to correlate with the condition of the ER [25]. Our results demonstrated that treatment with TiO_2_-NP decreased calnexin expression (Fig A and B in S3 Fig). These results show that TiO_2_-NP induce ER stress and affect the condition of the ER in human bronchial epithelial cells.

### TiO_2_ nanoparticles disrupt mitochondria by damaging MAMs and cellular calcium homeostasis

**Fig 3:**
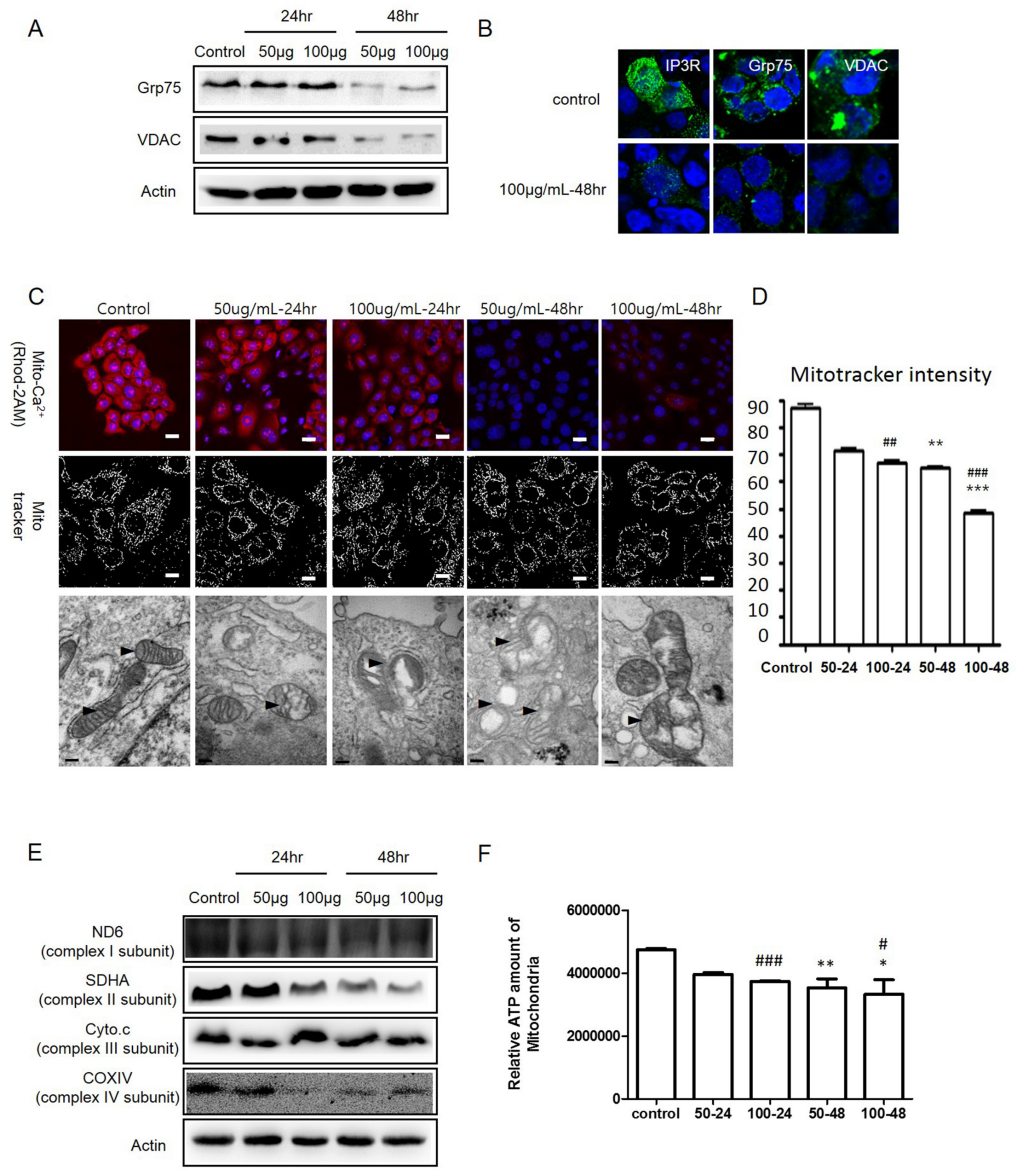
Titanium dioxide nanoparticles (TiO_2_-NP) damage the connection between the endoplasmic reticulum (ER) and mitochondria. (A) To detect disruption of the mitochondria-associated ER membranes (MAMs), proteins related to MAMs (Grp75 and VDAC) were detected using western blot analysis. (B) Immunofluorescence assay of IP3R, Grp75, and VDAC between the control and 100 μg/mL TiO_**2**_-NP treatment groups for 48 h. (Green, IP3R, Grp75, and VDAC; Blue, DAPI-Nucleus). (C) Upper panel, Ca^2+^ in the mitochondria was stained by Rhod-2 AM; middle panel, mitochondria staining with Mito tracker; bottom panel, TEM image of mitochondria after TiO_**2**_-NP treatment. (D) Mito tracker fluorescence of interest was analyzed further by the Mytoe program. Each bar represents mean ± SEM, n = 5. Significant differences are indicated by **p < 0.01, ***p < 0.001 (comparison between TiO_**2**_-NP treatment concentrations, 50 and 100 μg) and ^##^ *p* < 0.01, ^###^ *p* < 0.001 (comparison between TiO_**2**_-NP treatment time, 24 and 48 h). (E) To detect mitochondria dysfunction, the proteins related to the respiration chain in the mitochondria, including ND6 (complex subunit I), SDHA (complex subunit II), Cyto c (complex subunit III), and COXIV (complex subunit IV), were analyzed using western blot assay. (F) ATP levels in the mitochondria after treatment with TiO_**2**_-NP. Significant differences are indicated by *p < 0.05, **p < 0.01 (comparison between TiO_**2**_-NP treatment concentrations, 50 and 100 μg) and ^#^ *p* < 0.05, ^###^ *p* < 0.001 (comparison between TiO_**2**_-NP treatment times, 24 and 48 h). IP3R, inositol triphosphate receptor; VDAC1, voltage-dependent anion-selective channel protein 1; Grp75, 75 kDa glucose regulated protein; ND6, NADH dehydrogenase; SDHA, succinate dehydrogenase complex subunit A; Cyto C, cytochrome C; COXIV, cyto C oxidase IV.

Disruption of calcium homeostasis is related directly to the induction of ER stress [26]. To investigate Ca^2+^ homeostasis following TiO_2_-NP treatment, the integrity of mitochondria-associated ER membranes (MAMs), which regulate the Ca^2+^ flow between the ER and mitochondria, was evaluated. Disruption of MAMs was detected by measuring the expression levels of several cellular Ca^2+^-regulating proteins such as inositol triphosphate receptor (IP3R; a glycoprotein complex in the ER membrane), voltage-dependent anion-selective channel protein 1 (VDAC1; outer mitochondrial membrane protein that mediates Ca^2+^ flow), and 75-kDa glucose regulated protein (Grp75; a heat shock 70 family protein that regulates both IP3R and VDAC1) (Fig 3A and 3B and S4 Fig). To determine the quantitative changes in mitochondrial Ca^2+^ levels, cells were stained with Rhod-2 AM, a mitochondrial Ca^2+^ indicator. CLSM images revealed that the fluorescence decreased in TiO_2_-NP-treated cells. (Fig 3C, upper panel).

Changes in the mitochondrial status also were assessed using CLSM imaging with the Mito-tracker staining and TEM imaging. The Mito-tracker staining images showed that the mitochondria morphology was altered by TiO_2_-NP treatment; the alterations of the mitochondria were digitalized by the Mytoe program (Fig 3C, middle panel and Fig 3D). The Mytoe analysis revealed that the intensity of the Mito tracker staining decreased significantly in TiO_2_-NP-treated cells (Fig 3D). The TEM images also clearly demonstrated morphological changes in the mitochondria, such as swelling and disruption of the outer and inner mitochondrial membranes (Fig 3C, bottom panel). To determine the disruption of mitochondrial function, we measured the expression levels of the respiration chain-related proteins, NADH dehydrogenase (ND6, complex subunit I), succinate dehydrogenase complex subunit A (complex subunit II, SDHA), cytochrome C (Cyto C, complex subunit III), and cyto C oxidase IV (COX 1V, complex IV subunit). The western blot results showed that the protein levels decreased in TiO_2_-NP-treated cells (Fig 3E). To monitor mitochondrial biogenesis, the ATP levels were measured after treatment of cells with TiO_2_-NP. The cellular ATP levels decreased significantly (Fig 3F). Our results show that induced ER stress could demolish MAMs and mitochondrial calcium homeostasis in cells. In addition, induced ER stress could disrupt mitochondrial morphology and biogenesis.

### TiO_2_ nanoparticles induce autophagy in normal human lung cells

**Fig 4:**
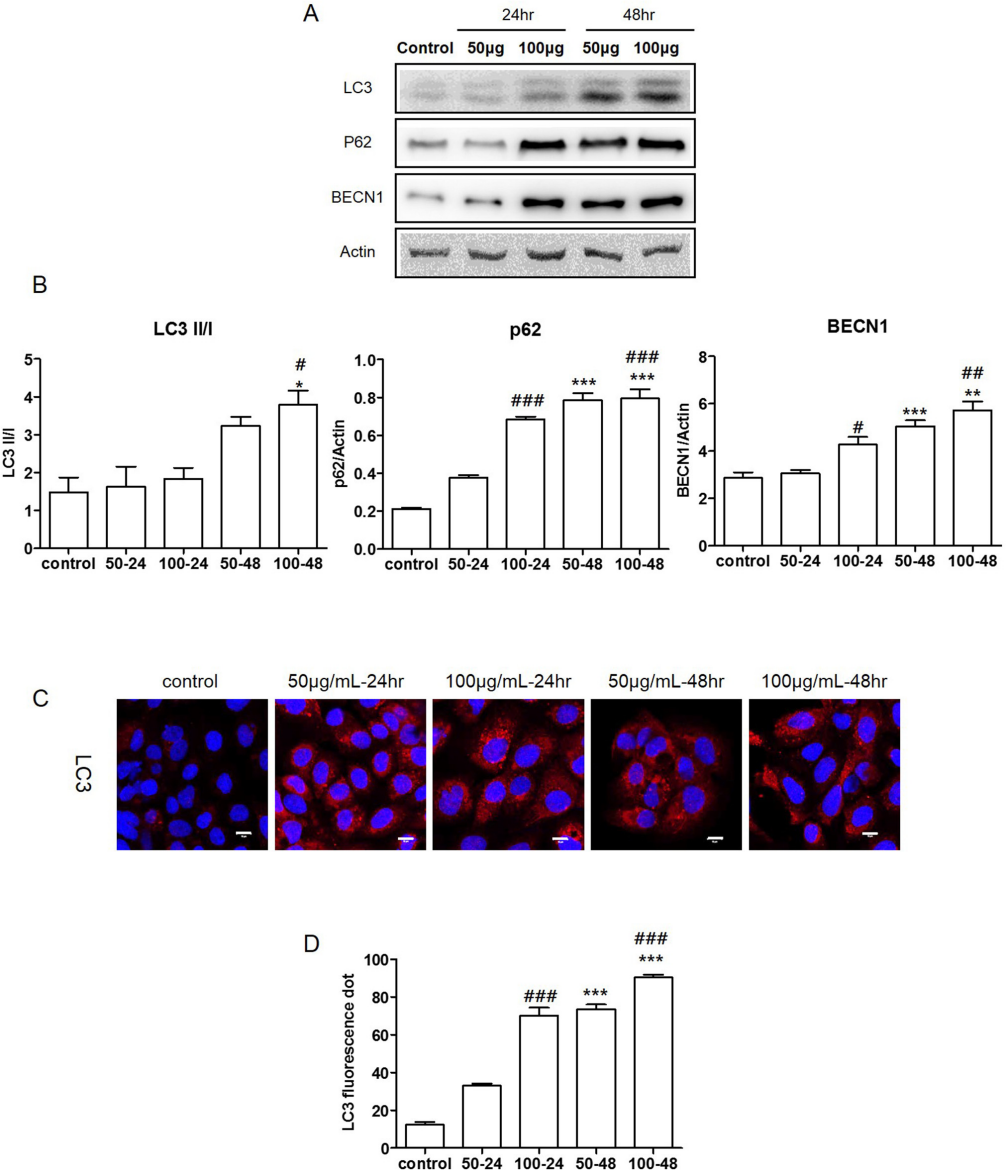
Titanium dioxide nanoparticles (TiO_2_-NP) induced autophagy in human bronchial epithelial cells. (A) Expression levels of the autophagy related proteins LC3, p62, and BECN1 were determined by western blot assay. (B) Each bar represents the mean ± SEM, n = 3, *p < 0.05, **p < 0.01, and ***p < 0.001 (comparison between TiO_**2**_-NP treatment concentrations, 50 and 100 μg), ^##^ *p* < 0.01 and ^###^ *p* < 0.001 (comparison between TiO_**2**_-NP treatment times, 24 and 48 h). (C) Immunofluorescence assay of LC3. (Red: LC3 and Blue: nucleus. Scale bar, 10 μm). (D) Increased numbers of red puncta and vacuoles counted in cells after TiO_**2**_-NP treatment. Each bar represents the mean ± SEM, n = 3, ***p < 0.001 (comparison between TiO_**2**_-NP treatment concentrations, 50 and 100 μg) ^###^ *p* < 0.001 (comparison between TiO_**2**_-NP treatment times, 24 and 48 h). LC3, microtubule-associated protein-1 light-chain 3; SQSTM1, p62, Sequestosome 1; BECN1, beclin-1.

ER stress-induced autophagy correlates with cell death [27]. To determine if autophagy was induced in TiO_2_-NP-treated cells, the expression levels of LC3, sequestosome-1 (SQSTM1/p62), and BECN1 were examined by western blot assay and densitometric analysis. LC3 is one of the key regulators of autophagy, which is measured quantitatively by the ratio of LC3 II to LC3 I [28]. Our results showed that the LC3 II/LC3 I ratio increased significantly in the TiO_2_-NP-treated cells. Additionally, the expression of SQSTM1/p62 and BECN1 also increased significantly in the TiO_2_-NP-treated cells (Fig 4A and 4B). Furthermore, the immunofluorescence assay revealed that TiO_2_-NP treatment increased the number of LC3 fluorescence puncta (red) (Fig 4C). We counted the LC3 puncta using the Image J software (Fig 4D). Taken together, our results clearly demonstrate that the TiO_2_-NP induces autophagy in human bronchial epithelial cells.

### Inhibition of ER stress decreases TiO_2_ nanoparticles cytotoxicity

**Fig 5:**
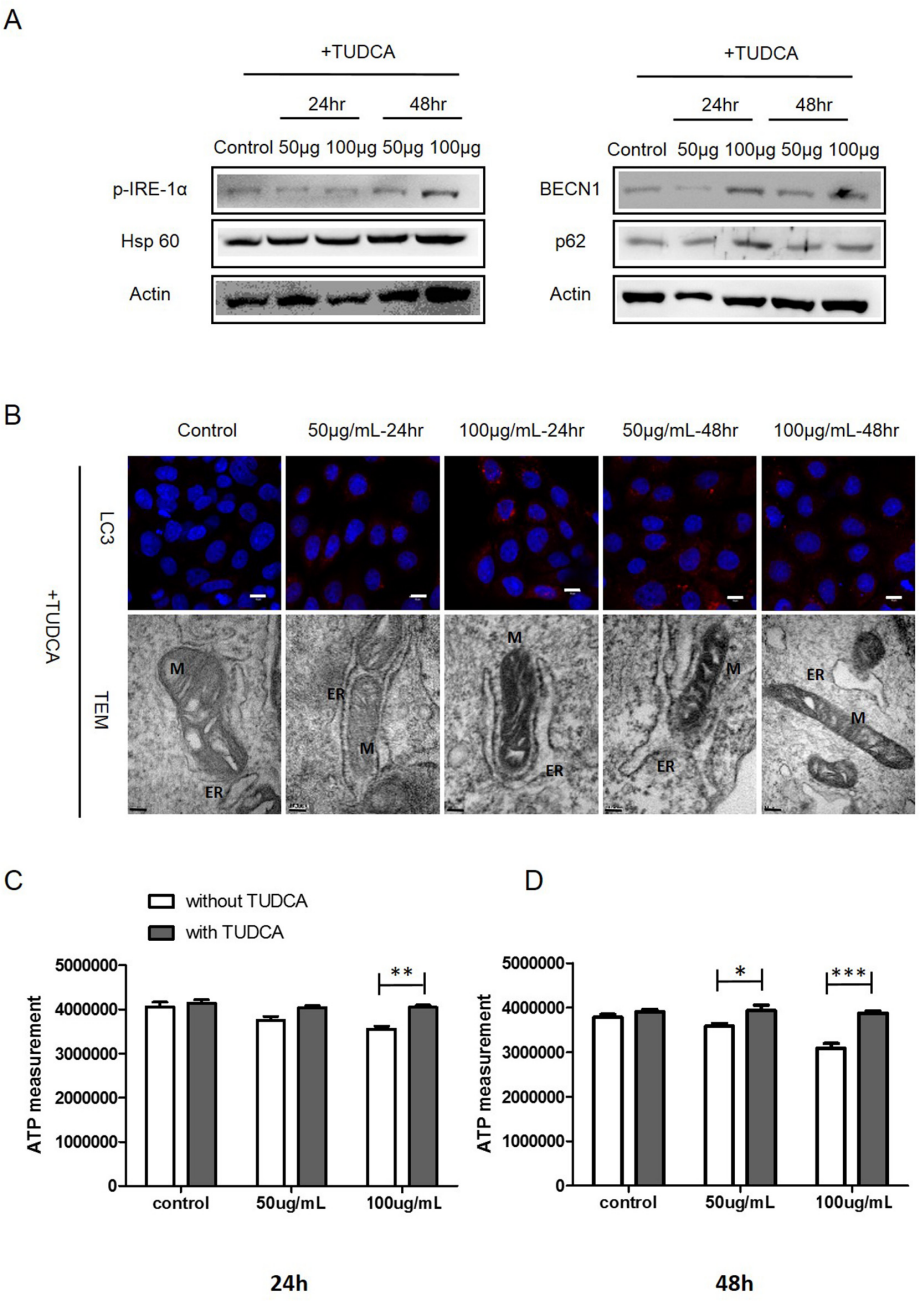
Treatment with the endoplasmic reticulum (ER) stress inhibitor (TUDCA) affects cell biogenesis. (A) Cells were treated with TUDCA followed by TiO_**2**_-NP, and then, western blot assay of p-IRE-1α, HSP60, BECN1, and p62 was performed. (B) LC3 levels were determined using immunofluorescence (scale bar, 10 μm). Mitochondria (M) and ER conditions were determined by TEM imaging. (C) Amount of ATP in cells after treatment with TiO_**2**_-NP with or without TUDCA for 24 h; ***P<0.001, **P<0.01, *p<0.05 (2-way ANOVA, Bonferroni's multiple comparison post-test), and (D) for 48 h (White bar, TiO_**2**_-NP only treatment; grey bar, TiO_**2**_-NP treatment with TUDCA). TUDCA, tauroursodeoxycholic acid; LC3, microtubule-associated protein-1 light-chain 3; HSP60, heat shock protein 60; p62, Sequestosome 1; BECN1, beclin-1; TEM, transmission electron microscopy.

Tauroursodeoxycholic acid (TUDCA) is a well-known inhibitor of ER stress [29]. To determine the potential effects of the inhibition of ER stress on mitochondria function disruption, autophagy, and cell toxicity, normal lung cells were treated with TUDCA (1mM) for 24h before exposure to TiO_2_-NP. Compared with the expression levels of actin, the internal standard, co-treatment with TiO_2_-NP and TUDCA did not induce any significant changes in the protein expression levels of phospho-IRE-1α, Hsp60, BECN, and p62 (Fig 5A). Furthermore, treatment with TUDCA and TiO_2_-NP did not cause any significant changes in the LC3 levels (Fig 5B). TEM images demonstrated no significant changes in the mitochondrial or ER morphology in cells pretreated with TUDCA (Fig 5B). To confirm whether ER stress is associated with the biogenesis of mitochondria, we compared the ATP levels of TiO_2_-NP-treated cells with TUDCA pretreatment with those without pretreatment. Our results indicated that TiO_2_-NP alone decreased ATP levels. However, TUDCA pretreatment prevented this effect (Fig 5C and 5D). The levels of ER marker, calnexin remained unaltered in TUDCA co-treated cells (S5 Fig). Together, our results indicate that the cells with blocked ER stress do not exhibit any autophagy and mitochondria disruption following treatment with TiO_2_-NP.

## Discussion

TiO_2_-NP are widely used owing to their unique characteristics, which include extended photostability, strong oxidizing energy, relatively low descriptive toxicity, and easy availability. Due to these properties, TiO_2_-NP are being used in diverse biomedical areas such as cell imaging, biosensors, genetic engineering in cancer therapy, drug delivery, and development of medical implants [5]. However, prior to its extensive use in humans, the mechanisms underlying the potential toxic effects of TiO_2_-NP should be carefully identified.

The ROS generated owing to nanomaterials, including metal oxide-based nanoparticles, TiO_2_-NP, carbon fullerenes, and carbon nanotubes, are associated closely with cell damage such as inflammation, oxidative stress, and cell organelle disruption [30–33]. Many studies have reported that TiO_2_-NP induce toxic response by increasing the generation of ROS, which disrupts redox homeostasis [34, 35]. Our H_2_-DCFDA staining data revealed the generation of ROS following TiO_2_-NP treatment in human bronchial epithelial cells (Fig 1 and S1 Fig). Therefore, we hypothesized that the elevated ROS levels affected the ER, which is one of the organelles that plays an important role in the cellular quality control systems and sensitivity to oxidative stress. In addition, this action may be a key mechanism of TiO_2_-NP toxicity. The ER is susceptible to diverse stressors such as misfolded proteins, environmental triggers, metabolic disturbances, and oxidative stress [36]. ER stress elevates Grp78/Bip, the glucose-regulated protein and chaperone [37]. The chaperone interacts with the misfolded proteins and promotes refolding, thereby playing a critical role in regulating three ER transmembrane proteins, protein kinase-like ER kinase (PERK), IRE-1α, and activating transcription factor 6 (ATF6) [38]. Diverse nanoparticles, including zinc oxide, silver nanoparticles, and iron oxide nanoparticles, were shown to elevate ER stress events [39, 40, 41]. However, only a few studies have shown that TiO_2_-NP could induce ER stress events in mammalian cells. Our data show that TiO_2_-NP could induce IRE-1α phosphorylation, elevate Grp78/Bip and CHOP, and activate the ER stress pathway in human bronchial epithelial cells. In addition, ER stress is strongly associated with the disruption of cellular Ca^2+^ homeostasis. The ER and mitochondria cooperatively regulate the cellular homeostatic network and act as physiological barriers against abnormal intracellular Ca^2+^ changes [42, 43]. Ca^2+^ translocates from the ER to the mitochondria through the MAMs. van Vliet et al. suggested that MAMs regulate energy metabolism, mitochondria biogenesis, and diverse signaling pathways involving ER stress and autophagy [15, 29]. To evaluate the effect of TiO_2_-NP on the MAMs, we evaluated specific proteins that have been identified in MAMs, including the IP3R, which plays a crucial role in Ca^2+^ handling in the ER, VDAC, and Grp75 [8, 44, 45]. Results of the fluorescence microscope imaging and western blot assay showed alterations in the MAM-related proteins following exposure to TiO_2_-NP (Fig 2A and 2B). ER stress decreases ER Ca^2+^ levels, which consequently reduces the expression of IP3R. IP3R functions as a transporter of Ca^2+^ from the ER to the mitochondria, and therefore, its decreased expression eventually diminishes the mitochondrial bioenergetics, leading to reduced cellular ATP production [46]. Furthermore, IP3R-mediated Ca^2+^ release from the ER is associated closely with increased levels of free cytoplasmic Ca^2+^, which activates autophagy [47]. Our data show that Ca^2+^ imbalance and abnormal autophagy increased following TiO_2_-NP exposure. In particular, decreased mitochondrial Ca^2+^ levels were detected with Rhod-2 AM staining in TiO_2_-NP-treated cells. The changes in the Ca^2+^ level affected the mitochondrial biogenesis disruption. In addition, we determined mitochondrial disorder by measuring the levels of proteins related to the respiration chain in the mitochondria and the ATP levels. To further support the correlation between ER stress and disruption of the MAMs, we treated cells with TUDCA, a bile acid that inhibits ER stress [29]. TUDCA pretreatment decreased ER stress and restored mitochondrial biogenesis and autophagy (Fig 5).

**Fig 6:**
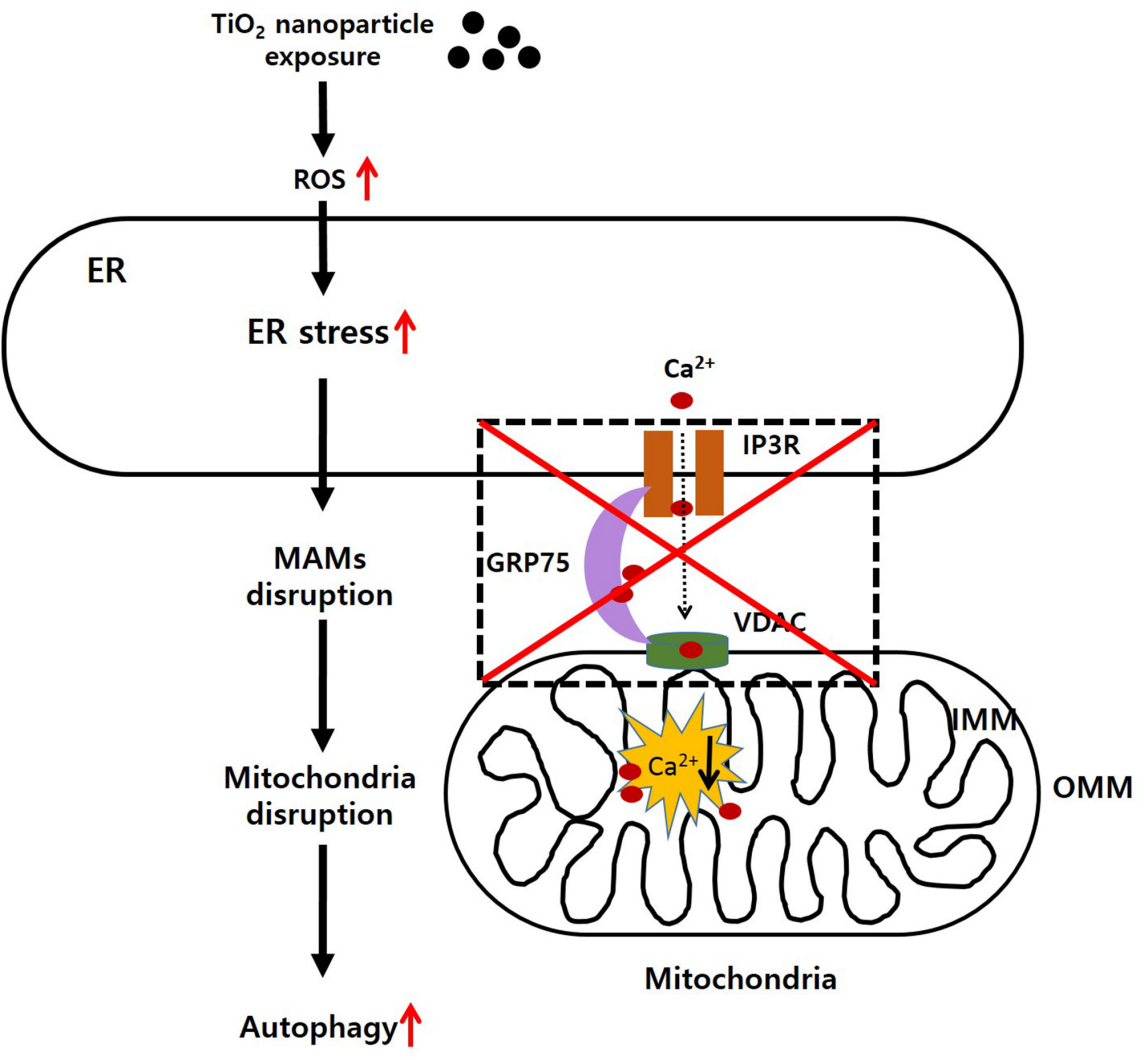
Schematic diagram of titanium dioxide nanoparticles (TiO_2_-NP) toxicity in human bronchial epithelial cells. Based on our study, exposure of cells to TiO_**2**_-NP generates reactive oxygen species (ROS), which causes endoplasmic reticulum (ER) stress. Induction of ER stress disrupts the mitochondria-associated ER membranes (MAMs) and causes mitochondrial calcium imbalance. Finally, autophagy is aberrantly induced and leads to cell death.

In conclusion, our data provide new evidence for the mechanism of TiO_2_-NP cytotoxicity, as demonstrated by increased ER stress effects, MAM disruption, dysfunction of mitochondria biogenesis, and autophagic cell death (Fig 6). The potential harmful effects of TiO_2_-NP nanoparticles have remained elusive. There are minimal regulatory guidelines from the government controlling their use for the industry. The safe widespread use of nanomaterials, including TiO_2_-NP, requires intensive study of their toxicities and mechanisms of action in relevant organisms. In future research, however, further works should be performed in order to discriminate whether observed results are due to the nanosized form or to the titanium component itself.

## Supporting Information

S1 Fig X-ray diffraction (XRD) data of titanium dioxide nanoparticles (TiO_2_-NP). XRD data of the rutile (red) and anatase (blue) forms of TiO_2_-NP and experimental materials (P-25, green).
(TIF)

S2 Fig Intracellular reactive oxygen species (ROS) detection and cell viability monitoring. After H_2_-DCFDA staining, we detected the fluorescence using confocal laser scanning microscopy. The green fluorescence intensity was calculated by Image J (Figure A). To assess the cell viability following TiO_2_-NP treatment, the MTT and WST-1 assays were performed. The optical densities of the samples were read on a microplate reader (Bio-Rad) at 450 nm. H_2_-DCFDA, 2,7-dichlorodihydrofluorescein diacetate (Figure B).
(TIF)

S3 Fig Measurement of calnexin in titanium dioxide nanoparticles (TiO_2_-NP)-treated cells. To monitor the state of the endoplasmic reticulum (ER), calnexin, an ER state marker, was detected. Western blot analysis of calnexin (ER marker) after TiO_2_-NP treatment (Figure A). Immunofluorescence assay with calnexin after TiO_2_-NP treatment; scale bar, 10 μm (Figure B).
(TIF)

S4 Fig Titanium dioxide nanoparticles (TiO_2_-NP) affect the mitochondria-associated endoplasmic reticulum (ER) membranes (MAMs). Immunofluorescence assay of IP3R (ER membrane, upper panel), Grp75 (middle panel), and VDAC1 (mitochondrial outer membrane, bottom panel; blue, DAPI-nucleus). IP3R, inositol triphosphate receptor; VDAC1, voltage-dependent anion-selective channel protein 1; Grp75, 75 kDa glucose regulated protein.
(TIF)

S5 Fig Effect of TUDCA in titanium dioxide nanoparticles (TiO_2_-NP) treated cells. To monitor the state of the endoplasmic reticulum (ER) after TUDCA treatment, calnexin, an ER state marker, was detected. Immunofluorescence assay of calnexin after TUDCA (ER stress inhibitor) treatment and with TiO_2_-NP; green, calnexin; blue, DAPI-nucleus. TUDCA, tauroursodeoxycholic acid.
(TIF)
