# Supplementary figures and images for: Titanium Dioxide Nanoparticles Induce Endoplasmic Reticulum Stress-Mediated Autophagic Cell Death via Mitochondria-Associated Endoplasmic Reticulum Membrane Disruption in Normal Lung Cells
Source: PLoS One. 2015 Jun 29;10(6):e0131208. doi: 10.1371/journal.pone.0131208 (PMC4485469; doi:10.1371/journal.pone.0131208)

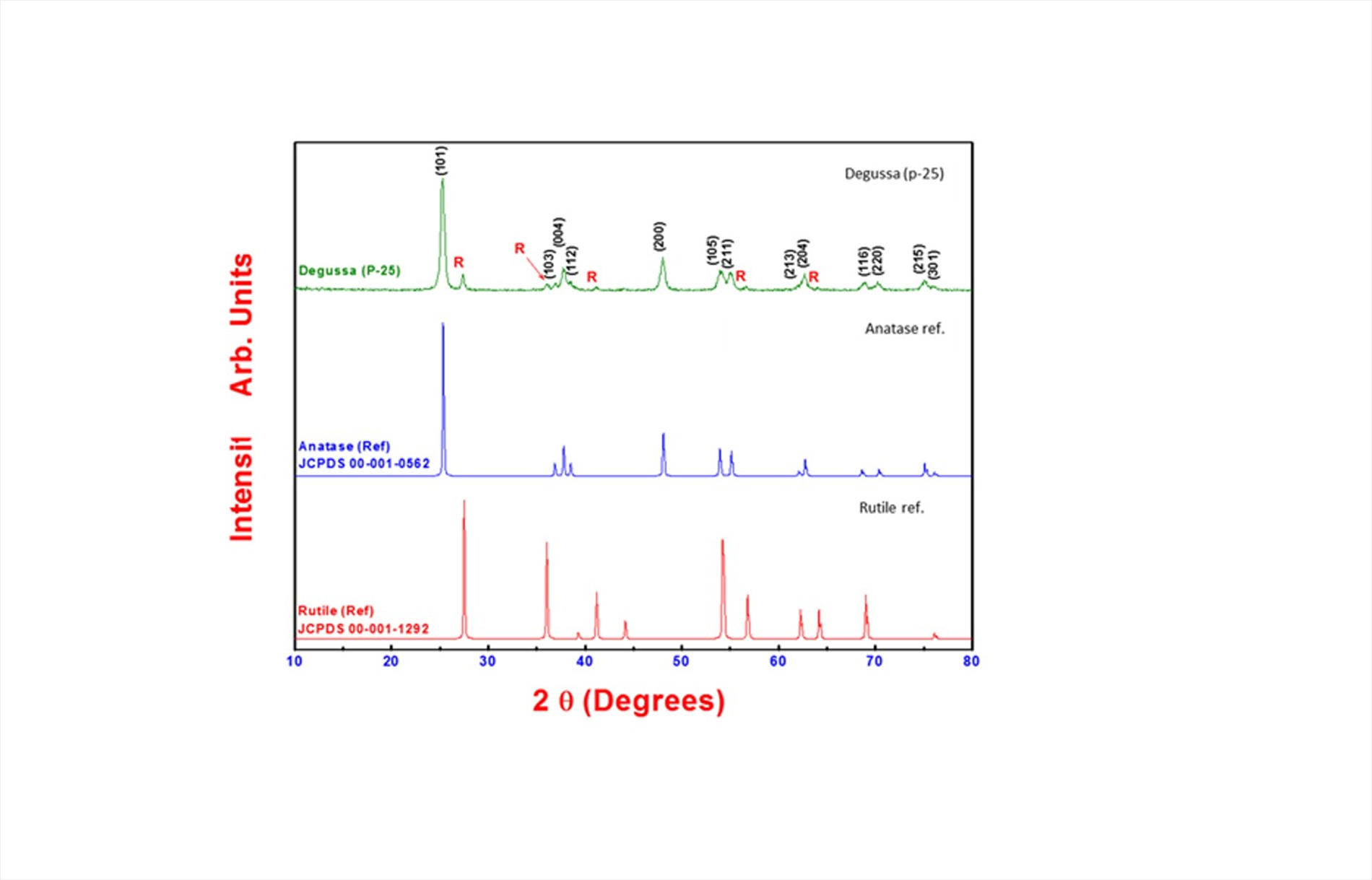

Supplement: S1 Fig — XRD data of the rutile (red) and anatase (blue) forms of TiO2-NP and experimental materials (P-25, green). (TIF) [file pone.0131208.s001.tif]

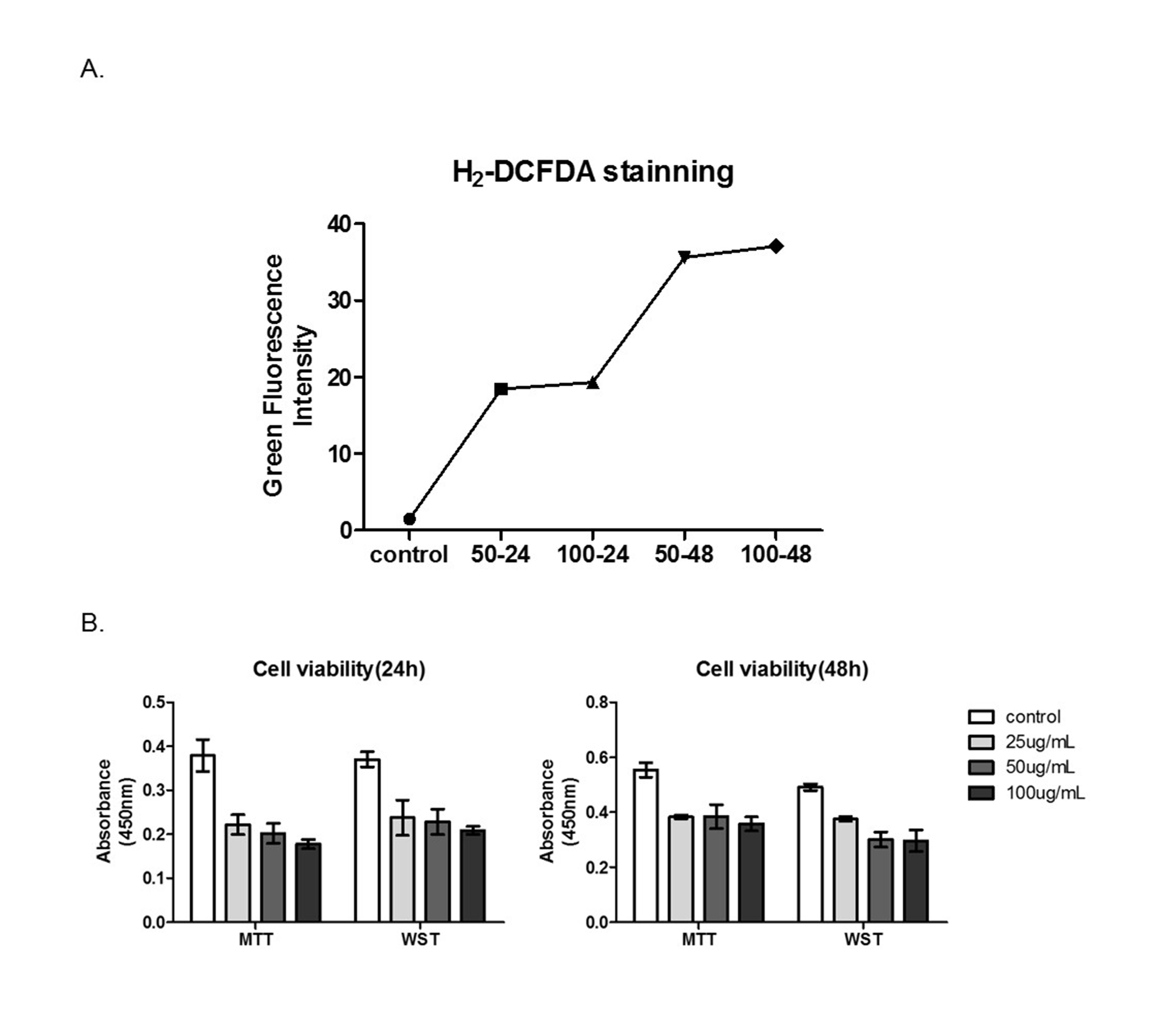

Supplement: S2 Fig — After H2-DCFDA staining, we detected the fluorescence using confocal laser scanning microscopy. The green fluorescence intensity was calculated by Image J (Figure A). To assess the cell viability following TiO2-NP treatment, the MTT and WST-1 assays were performed. The optical densities of the samples were read on a microplate reader (Bio-Rad) at 450 nm. H2-DCFDA, 2,7-dichlorodihydrofluorescein diacetate (Figure B). (TIF) [file pone.0131208.s002.tif]

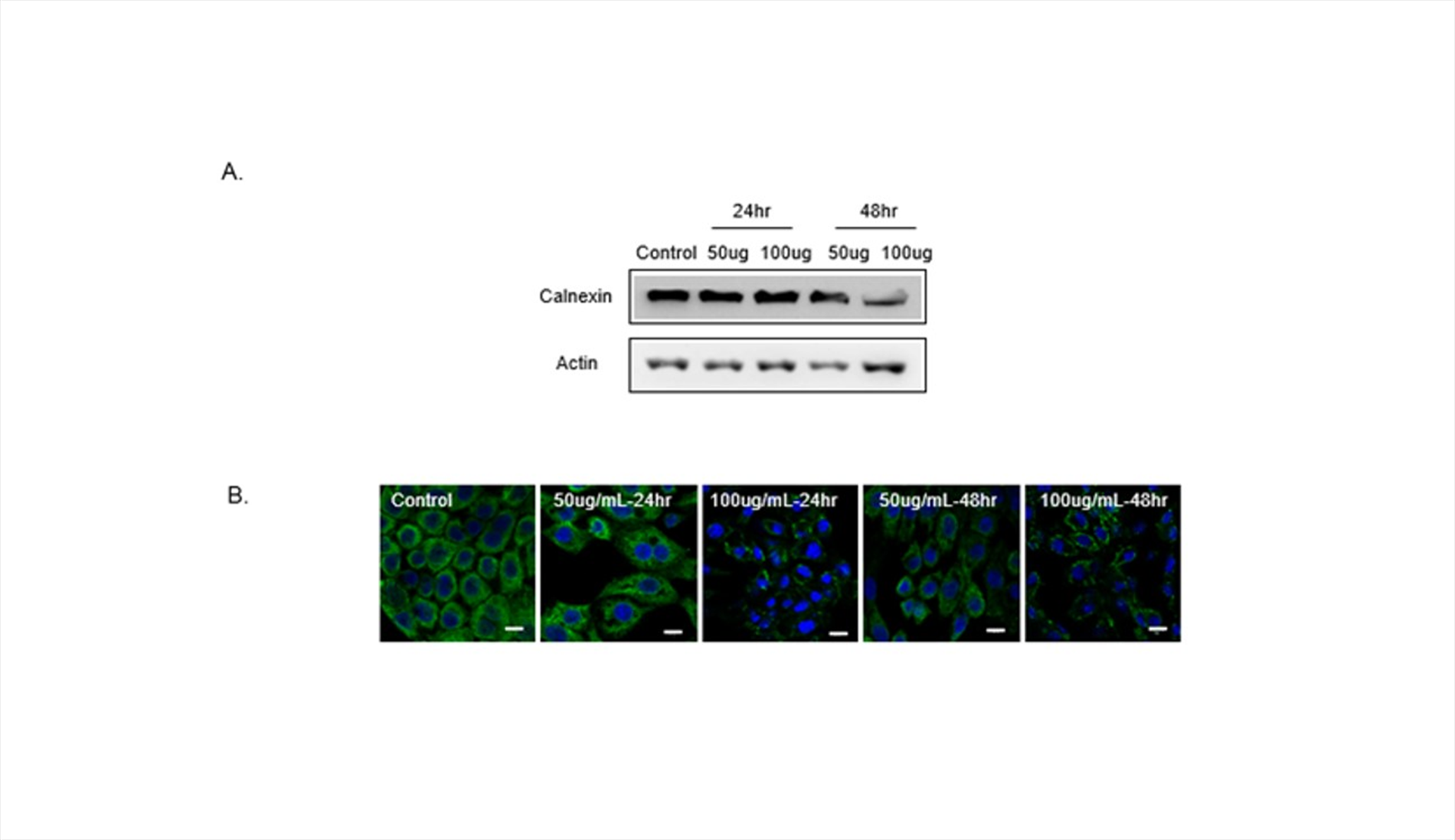

Supplement: S3 Fig — To monitor the state of the endoplasmic reticulum (ER), calnexin, an ER state marker, was detected. Western blot analysis of calnexin (ER marker) after TiO2-NP treatment (Figure A). Immunofluorescence assay with calnexin after TiO2-NP treatment; scale bar, 10 μm (Figure B). (TIF) [file pone.0131208.s003.tif]

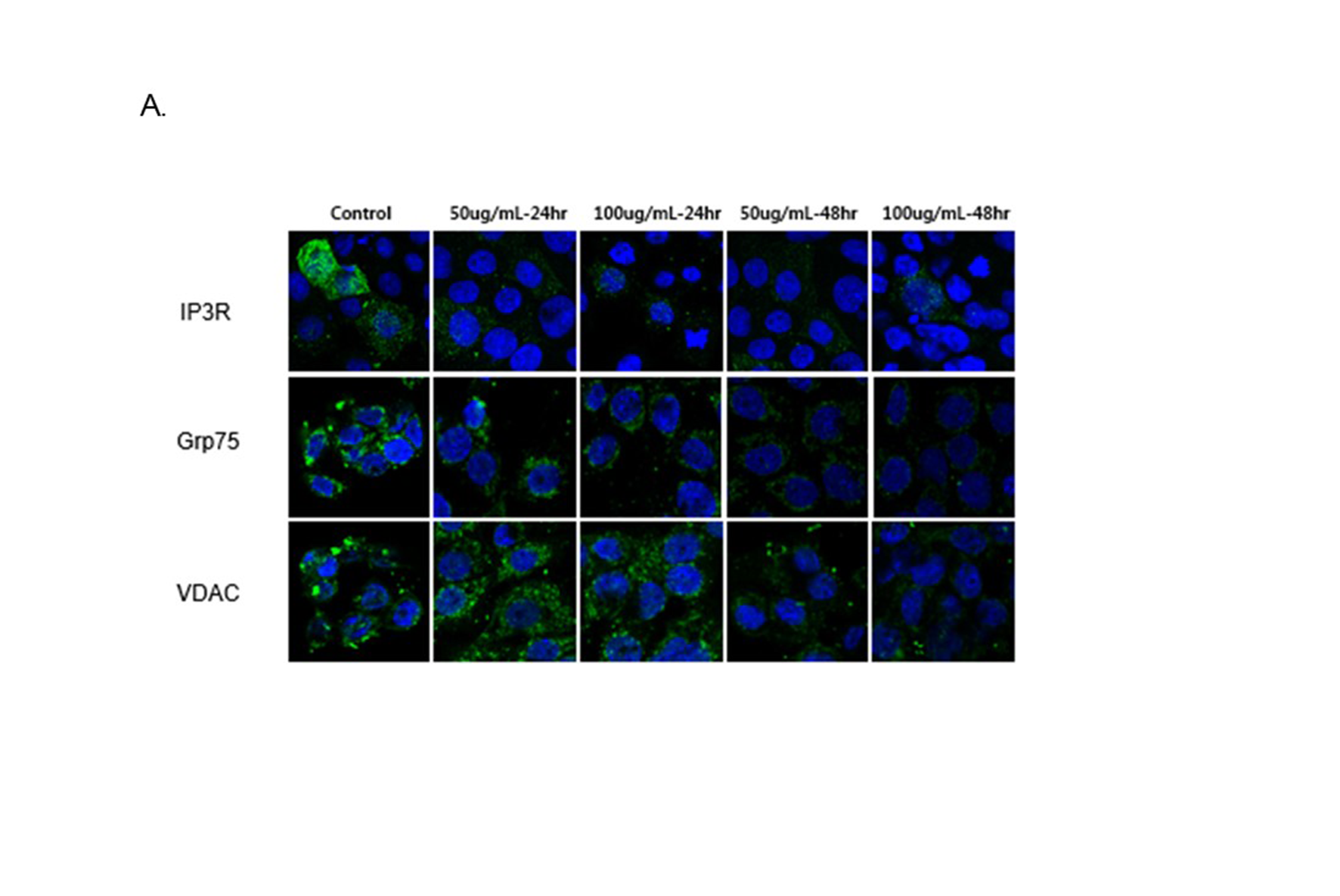

Supplement: S4 Fig — Immunofluorescence assay of IP3R (ER membrane, upper panel), Grp75 (middle panel), and VDAC1 (mitochondrial outer membrane, bottom panel; blue, DAPI-nucleus). IP3R, inositol triphosphate receptor; VDAC1, voltage-dependent anion-selective channel protein 1; Grp75, 75 kDa glucose regulated protein. (TIF) [file pone.0131208.s004.tif]

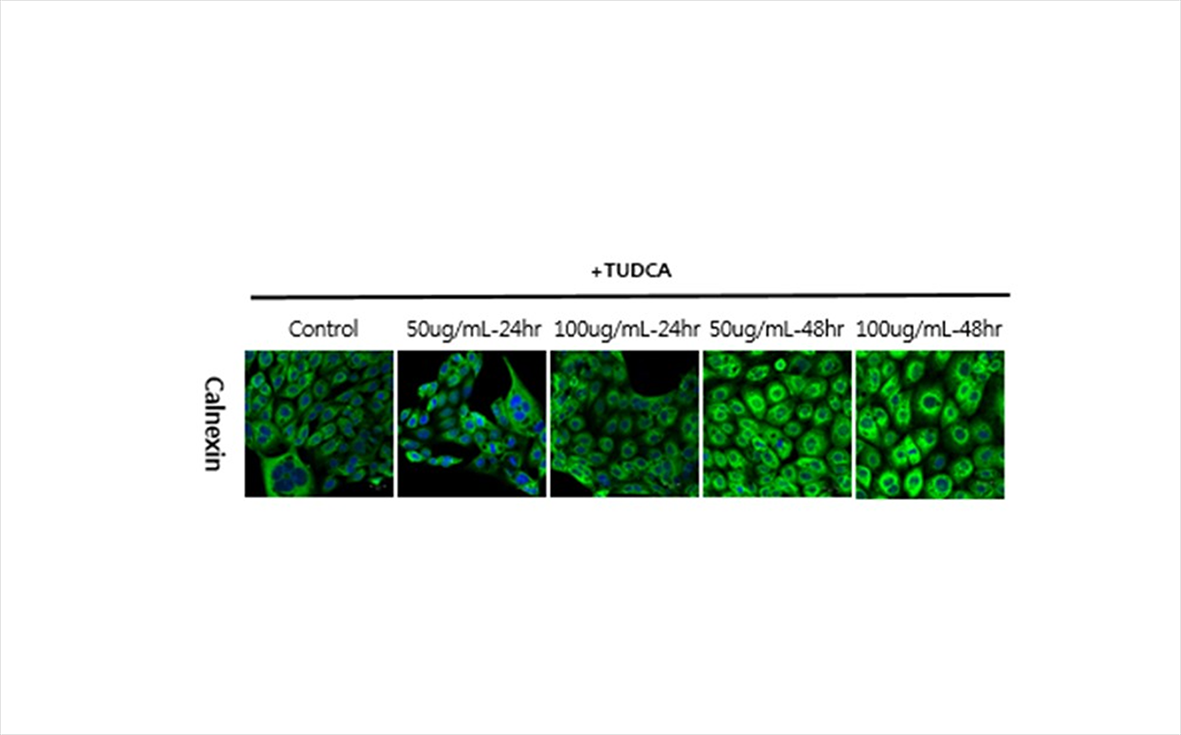

Supplement: S5 Fig — To monitor the state of the endoplasmic reticulum (ER) after TUDCA treatment, calnexin, an ER state marker, was detected. Immunofluorescence assay of calnexin after TUDCA (ER stress inhibitor) treatment and with TiO2-NP; green, calnexin; blue, DAPI-nucleus. TUDCA, tauroursodeoxycholic acid. (TIF) [file pone.0131208.s005.tif]
